# Brain‐wide inferiority and equivalence tests in fMRI group analyses: Selected applications

**DOI:** 10.1002/hbm.25664

**Published:** 2021-09-16

**Authors:** Martin Fungisai Gerchen, Peter Kirsch, Gordon Benedikt Feld

**Affiliations:** ^1^ Department of Clinical Psychology Central Institute of Mental Health, Medical Faculty Mannheim, Heidelberg University Mannheim Germany; ^2^ Bernstein Center for Computational Neuroscience Heidelberg/Mannheim Mannheim Germany; ^3^ Department of Psychology Heidelberg University Heidelberg Germany; ^4^ Department of Addiction Behavior and Addiction Medicine Central Institute of Mental Health, Medical Faculty Mannheim, Heidelberg University Mannheim Germany; ^5^ Department of Psychiatry and Psychotherapy Central Institute of Mental Health, Medical Faculty Mannheim, Heidelberg University Mannheim Germany

**Keywords:** confidence interval, equivalence tests, functional magnetic resonance imaging, Hedge's *g*, null hypothesis significance testing

## Abstract

Null hypothesis significance testing is the major statistical procedure in fMRI, but provides only a rather limited picture of the effects in a data set. When sample size and power is low relying only on strict significance testing may lead to a host of false negative findings. In contrast, with very large data sets virtually every voxel might become significant. It is thus desirable to complement significance testing with procedures like inferiority and equivalence tests that allow to formally compare effect sizes within and between data sets and offer novel approaches to obtain insight into fMRI data. The major component of these tests are estimates of standardized effect sizes and their confidence intervals. Here, we show how Hedges' *g*, the bias corrected version of Cohen's *d*, and its confidence interval can be obtained from SPM *t* maps. We then demonstrate how these values can be used to evaluate whether nonsignificant effects are really statistically smaller than significant effects to obtain “regions of undecidability” within a data set, and to test for the replicability and lateralization of effects. This method allows the analysis of fMRI data beyond point estimates enabling researchers to take measurement uncertainty into account when interpreting their findings.

## Graphical abstract

Inferiority and equivalence tests allow to compare effect sizes within and between data sets and are based on standardized effect sizes and their confidence intervals. We show how Hedges'*g* and its confidence interval can be obtained from SPM t maps and demonstrate how these can be used to obtain “regions of undecidability” within a data set, to test for the replicability, and for the lateralization of effects.

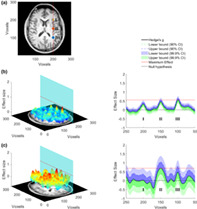

## 1 INTRODUCTION

Functional magnetic resonance imaging (fMRI) relies heavily on statistical analyses to draw inferences and the use of null hypothesis significance testing (NHST) is the major statistical approach in the field. A major downside of the NHST framework is that it does not emphasize the comparison of effects, but rather pits a point null hypothesis against all alternatives, and thus provides only a rather restricted picture of the effects in a data set.

Without a priori constraints the large number of voxels that are recorded in fMRI inevitably leads to a multiple testing problem that is addressed by applying conservative corrections to the critical value and other methods for type I error control. However, this stringent correction can lead to studies that have low power (i.e., large type II error), especially when assuming weak distributed effects (Cremers, Wager, & Yarkoni, 2017). This is especially critical since small sample sizes tend to overestimate effect sizes in clusters passing the significance threshold, which leads to unreasonable expectations towards what constitutes a meaningful effect in fMRI research (Button et al., 2013; Ioannidis, 2008; Lindquist & Mejia, 2015; Reddan, Lindquist, & Wager, 2017). This problem is especially critical when trying to replicate findings.

In contrast, with a very high number of participants an analysis will in most cases deliver a large number of significant voxels. In most cases it is also conceptually more interesting to know whether the activation of a brain region is more or less strongly associated with a specific behavior or intervention (see e.g., Bowring, Telschow, Schwartzman, & Nichols, 2019; Bowring, Telschow, Schwartzman, & Nichols, 2021).

**FIGURE 1:**
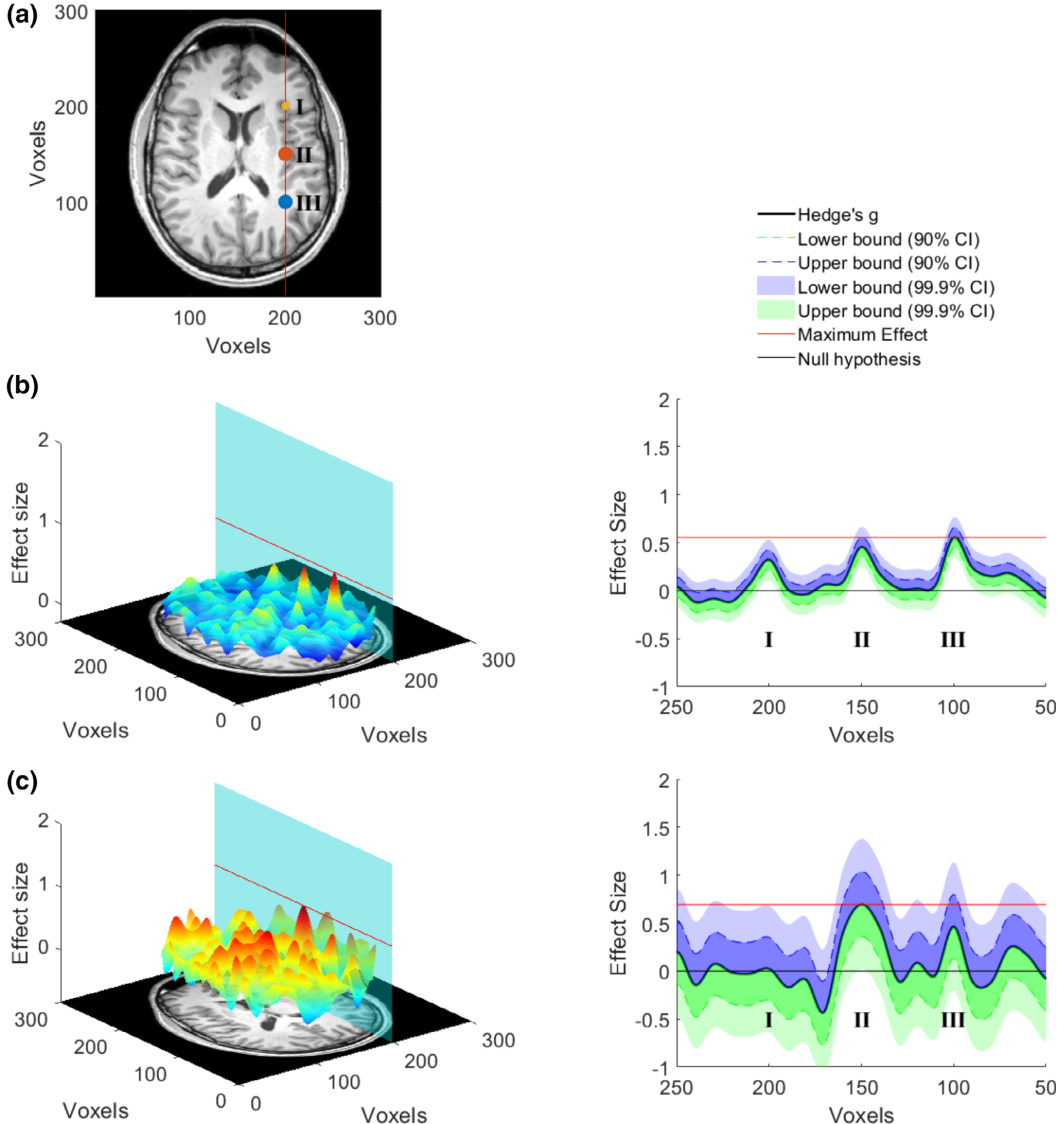
Data simulation. (a) Three effects located at I, II, and III (*d* = 0.28, *d* = 0.50, and *d* = 0.50, respectively) were generated for one fMRI‐slice in a simulated dataset that compared two conditions (two sample *t*‐test, see supplement for details). (b) The left panel shows the effect size per voxel for a large sample (*n* = 500 per group) drawn from the simulated population. The plane cuts the 3‐d graph at voxel 200, where the effects were inserted, and the red line marks the maximum effect size. In the right panel, all effect sizes lying on the plane are shown with the 90% and 99.9% CI added. The lower bound of the 99.9% CI in location I, II, and III is above 0 indicating that these voxels would be significant in an uncorrected whole‐brain *t*‐test with α = 0.001. This means that in a large sample all three effects that were inserted into the data can be recovered. In addition, the maximum effect size (red line, effect at III) is larger than the 90% CI of the effect located I, which following the logic of equivalence testing, would enable to conclude that the effect in I is smaller than the effect in III. The same is not true for effects III and II as the red line cuts the 90% CI of effect II. (c) This panel shows the same as (b) however of a smaller sample (*n* = 50 per group). On the left it is evident that the effect sizes that are being estimated are much noisier, which is a result of the smaller sample size. On the right side it is evident that the CIs are also much enlarged, showing that the point estimate of the effect is much more uncertain. Consequently, only the effect in II is significant at the whole‐brain threshold (*p* < .001). Importantly, we are also able to determine that most other voxels on this plane have effects that are smaller than the maximum effect found in the significant cluster of voxels. However, there is a cluster of voxels that are not significantly different from 0 at III, but that can also not be determined to be smaller than the effect present at II. Since the ground truth of the simulation is known this makes sense. Our method enables to identify such clusters in the whole brain and thereby allows deciding which brain areas can be excluded from being a relevant driver of certain behaviors and which cannot. Of course, the chosen threshold (peak voxel in this simulated case) will strongly influence the interpretation. Please see our use cases for indications on useful thresholds

To harness such information, it has been suggested for some time to supplement thresholded statistical parametric maps from NHST with maps of effect sizes (ES) (Jernigan, Gamst, Fennema‐Notestine, & Ostergaard, 2003). However, rather than relying just on point estimates for ESs, the construction of their confidence intervals (CIs) provides the means to conduct more formal equivalence testing (Lakens, Scheel, & Isager, 2018; see Figure 1 for an example explaining our approach using simulated data). Equivalence tests are able to show whether the data suggest that there is no effect larger (in absolute terms) than a specified threshold, the equivalence threshold. This can be achieved by using two one‐sided tests, that is, the first one‐sided test is calculated against the positive equivalence threshold and the other one‐sided test is calculated against the negative equivalence threshold. If both tests are significant, the measured effect is assumed to be between the two equivalence bounds. If only one of the bounds is used for testing, it is an inferiority test, as the procedure establishes that the effect is smaller than this bound. Importantly, instead of calculating one‐sided tests against the equivalence bounds, a 90% confidence interval (CI) around the measured effect size can be calculated and equivalence is established if this confidence interval only contains values between the equivalence bounds. For a more detailed explanation of the procedures in equivalence and inferiority testing see for example, Lakens et al. (2018), Schuirmann (1987), Walker and Nowacki (2011) and Wellek (2010).

Such approaches have so far rarely been applied to MRI data (see e.g., Bowring et al., 2021; Pardoe et al., 2016; Reggev, Brodie, Cikara, & Mitchell, 2020). With this paper we want to contribute to the use of this important statistical method in fMRI research. As a step towards this goal, we demonstrate here how Hedges' g, the bias‐corrected version of Cohen's *d*, and its CI can be obtained using brain‐wide t‐maps from group analyses with one‐sample and two‐sample *t*‐tests in statistical parametric mapping (SPM) with relative ease. We then provide concrete use cases that highlight how different inferiority and equivalence bounds allow different types of relevant inferences in fMRI data. We think that these procedures can provide a tool to further capture the richness of information in fMRI data and foster our understanding of brain processes.

## 2 METHODS

In this section, we first discuss standardized ES and their CIs for *t*‐tests in general and then specifically for fMRI group analyses.

### 2.1 Standardized effect sizes for *t*‐tests

One of the best known “families” of standardized ES is the “*d*”‐family where a mean difference is standardized by a respective standard deviation:

(1) $$d=\frac{m_{2}-m_{1}}{s}$$

with subscripts referring to experimental groups. Different “flavors” of *d* exist that differ in the exact estimation of the standard deviation. One specifically useful form for estimating effect sizes is based on the pooled standard deviation *s*_*p*_

(2) $$\begin{array}{c} s_{p}=\sqrt{\frac{\left(n_{1}-1\right)s_{1}^{2}+\left(n_{2}-1\right)s_{2}^{2}}{n_{1}+n_{2}-2}} \end{array}$$

which gives *d*_*p*_

(3) $$\begin{array}{c} d_{p}=\frac{m_{2}-m_{1}}{s_{p}} \end{array}$$

Please note that *d*_*p*_ is a version of Cohen's *d*, but is also sometimes called Cohen's *g* or Hedges'*g*. Here, we keep to the suggestion of Cohen (1988) to use subscripts for naming (see also Lakens, 2013). *d*_*p*_ is closely related to the *t*‐test and can be directly calculated from *t* values by:

(4) $$\begin{array}{c} d_{p}=t\sqrt{\frac{1}{n_{1}}+\frac{1}{n_{2}}} \end{array}$$

in the case of a two‐sample *t*‐test (Equation [16.21] in Rosenthal (1994); Equation [2] in Hentschke and Stüttgen (2011); Nakagawa & Cuthill (Nakagawa & Cuthill, 2007), and equivalently by:

(5) $$\begin{array}{c} d_{p}=t\sqrt{\frac{1}{n}} \end{array}$$

in the case of a one‐sample *t*‐test (see e.g., Bossier, Nichols, & Moerkerke, 2019, p. 16).

*d*_*p*_ is, however, a biased estimate of the population effect size, especially when it is based on a small sample size of *n* < 20 per group, and needs to be corrected by a correction factor *J* (Hedges, 1981; Hedges & Olkin, 1985) to provide the unbiased effect size Hedge's *g*

(6) $$\begin{array}{c} g=d_{p}\times J \end{array}$$

(Hedges, 1981) provides the following approximation to *J* by

(7) $$\begin{array}{c} J\approx \left(1-\frac{3}{4\mathrm{DoF}-1}\right) \end{array}$$

where DoF are the degrees of freedom used to estimate *s*.

CIs for ES can be estimated by bootstrap, exact analytical, or approximate analytical procedures (Hentschke & Stüttgen, 2011). Bootstrap and exact analytical procedures are computational much more demanding, while approximate analytical procedures are quite fast, but not always available (see Hentschke & Stüttgen, 2011). For the two‐sample *t*‐test an approximate analytical procedure is available which provides the standard error for the calculation of the CI of *g*

(8) $$\begin{array}{c} {\mathrm{se}}_{g}=\sqrt{\frac{n_{1}+n_{2}}{n_{1}n_{2}}+\frac{g^{2}}{2\left(n_{1}+n_{2}-2\right)}} \end{array}$$

(Nakagawa & Cuthill, 2007, Equation [17] in table 3).

In all cases, also when no approximate analytical procedure exists, CIs can be estimated by an exact analytical procedure based on the noncentral *t*‐distribution (Cumming & Finch, 2001; Smithson, 2003, p. 34; Steiger & Fouladi, 1997). This procedure uses computationally intensive routines to estimate the CI for the noncentrality parameter Δ of the noncentral *t*‐distribution with Δ = *t* and the respective DoF. Because the cumulative distribution function of the noncentrality parameter is strictly increasing and monotonic, and the effect size is a monotonic, strictly increasing continuous function of this function, the obtained lower and upper limit Δ_*l*_ and Δ_*u*_ of the noncentrality parameter CI can directly be inverted to the limits of the CI of the ES *g* by

(9) $$\begin{array}{c} {\mathrm{CI}}_{g}=\left[\Delta_{l}\sqrt{\frac{n_{1}+n_{2}}{n_{1}n_{2}},}\;\Delta_{u}\sqrt{\frac{n_{1}+n_{2}}{n_{1}n_{2}},}\right]=\left[\Delta_{l}\sqrt{\frac{1}{n_{1}}+\frac{1}{n_{2}}},\;\Delta_{u}\sqrt{\frac{1}{n_{1}}+\frac{1}{n_{2}}}\right] \end{array}$$

for the two‐sample *t*‐test (Smithson, 2003, Equation [4.7]) and by

(10) $$\begin{array}{c} {\mathrm{CI}}_{g}=\left[\Delta_{l}\sqrt{\frac{1}{n}},\;\Delta_{u}\sqrt{\frac{1}{n}}\right] \end{array}$$

for the one‐sample *t*‐test (Smithson, 2003, Equation [4.4]). Please see Steiger and Fouladi (1997) and Cumming and Finch (2001) for comprehensive explanations of the procedure.

The limits of the CI of the ES are estimated based on the empirical test statistic t and thus similar values would be obtained with this procedure for Cohen's *d* and Hedges'g. It is however important to note that the CI limits do not require bias correction and that Hedges'g is the unique unbiased estimator of δ for which the CI is valid (Hedges, 1981). Because of the correspondence between classical NHST and the ES CI where a significant result is equivalent to the CI not including 0, applying bias correction to the CI limits or recalculating t from Hedges'g would mean that either the criteria for statistical testing or the test statistic need to be bias corrected. This is however not the case because the test distribution for significance testing and the noncentral t distribution for CI estimation are chosen with the correct DoF. Thus, only the Cohen's *d* point estimator does not take the DoF into account, and needs to be corrected by Equation (7).

For estimating the limits of the CI of the noncentrality parameters we use the “ncpci.m” function of the Measures of Effect Size Matlab toolbox (Version 1.6.1; https://github.com/hhentschke/measures-of-effect-size-toolbox) by Hentschke and Stüttgen (2011).

### 2.2 Standardized effect sizes for *t*‐tests in fMRI

In fMRI analyses, however, *t*‐tests are usually implemented in a general linear model (GLM) approach in which specific contrasts are tested for significance. Fortunately, the procedures based on “the noncentral *t*‐distribution can be used to obtain confidence intervals for the standardized effect‐size measure Cohen's *d* in any situation where a t test is legitimate” (Smithson, 2003, p. 62). However, unlike standard *t*‐tests, in the GLM imaging analyses additional covariates, for example to correct for age and sex, are commonly included in the model and thus have to be taken into account when the procedures described above should be applied.

In SPM *t*‐tests are implemented by:

(11) $$\begin{array}{c} t=\frac{c^{\prime }b}{\sqrt{s^{2}c^{\prime }{\left(X^{\prime }X\right)}^{-}c}} \end{array}$$

where *X* is the (pre‐whitened and filtered) design matrix, *c* the contrast vector, *b* the estimated regression coefficients, *c*' the transpose of *c*, (*X*'*X*)^−^ the pseudoinverse of *X*'*X*, and *s* ^2^ the residual variance (see e.g., Penny, Friston, Ashburner, Kiebel, & Nichols, 2011, Equation [8.12]). *s* ^2^ is given by

(12) $$\begin{array}{c} s^{2}=\frac{e^{\prime }e}{\mathrm{DoF}}\; \end{array}$$

where *e*'*e* are the residual sum of squares and DoF = *N*‐*p* where *N* is the number of samples (i.e., the number of rows of *X*) and *p* is the rank of *X*. These DoF are used in SPM to test for the significance of *t*.

The ES *d* for a specific contrast c in this case would have the form of

(13) $$\begin{array}{c} d=\frac{c^{\prime }b}{s} \end{array}$$

From Equations (11) and (13), it follows that

(14) $$\begin{array}{c} d=\frac{c^{\prime }b}{s\sqrt{c^{\prime }{\left(X^{\prime }X\right)}^{-}c}}\sqrt{c^{\prime }{\left(X^{\prime }X\right)}^{-}c}=t\sqrt{c^{\prime }{\left(X^{\prime }X\right)}^{-}c} \end{array}$$

See also Bowring et al. (2021). Please note that for conventional one‐sample *t*‐tests $\sqrt{c\prime {\left(X\prime X\right)}^{-}c}=\sqrt{\frac{1}{n}}$ and DoF = *n* − 1, and for conventional two‐sample *t*‐tests $\sqrt{c\prime {\left(X\prime X\right)}^{-}c}=\sqrt{\frac{1}{n_{1}}+\frac{1}{n_{2}}}$ and DoF = *n*_1_ − 1 + *n*_2_ − 1. In the case that covariates are added to the model, the DoF are decreased by the number of covariates and $\sqrt{c\prime {\left(X\prime X\right)}^{-}c}$ will take into account correlations of the covariates with the regressors included in the contrast.

Bias correction depends on the DoF and can be conducted in this case as in Equation (6)

g=d×J

with the respective DoF entered in Equation (7). Also, the estimation of the limits of the CI of the noncentrality parameter of the noncentral *t*‐distribution depends on the DoF and can be conducted accordingly. The limits of the CI of the noncentrality parameter can then be converted to the limits of the CI of *g* by

(15) $$\begin{array}{c} {\mathrm{CI}}_{g}=\left[\Delta_{l}\sqrt{c^{\prime }{\left(X^{\prime }X\right)}^{-}c},\;\Delta_{u}\sqrt{c^{\prime }{\left(X^{\prime }X\right)}^{-}c}\right] \end{array}$$

The script to estimate ES and their CI from SPM *t* maps is available on Github at https://github.com/Fungisai/g_ci_spm.

## 3 RESULTS

In this section, we provide results for selected examples to demonstrate how the described methods can be used to obtain further insight into fMRI data. All participants were fully informed about the procedures and provided written informed consent.

### 3.1 Within‐sample comparison: “maps of undecidability”

Relying overly on NHST to separate activated from not activated brain regions in an (underpowered) fMRI study provides a distorted picture of the present effects. Here, we suggest a formal strategy to address the question, which brain areas have ES that are statistically indistinguishable from the effects in a detected cluster above the statistical threshold. The resulting maps of such an analysis identify areas, which are “empty” in NHST, but where the suggestive conclusion of a smaller ES than in a detected cluster is not valid. Therefore, we call the obtained results “maps of undecidability”. More technically speaking, we test in every voxel whether the upper bound of its ES 90% CI is including or exceeding a reference ES representative for a detected cluster. Obviously, the results depend on the selected representative ES. In our example, we use the voxel with the median ES in the detected clusters as the reference.

**FIGURE 2:**
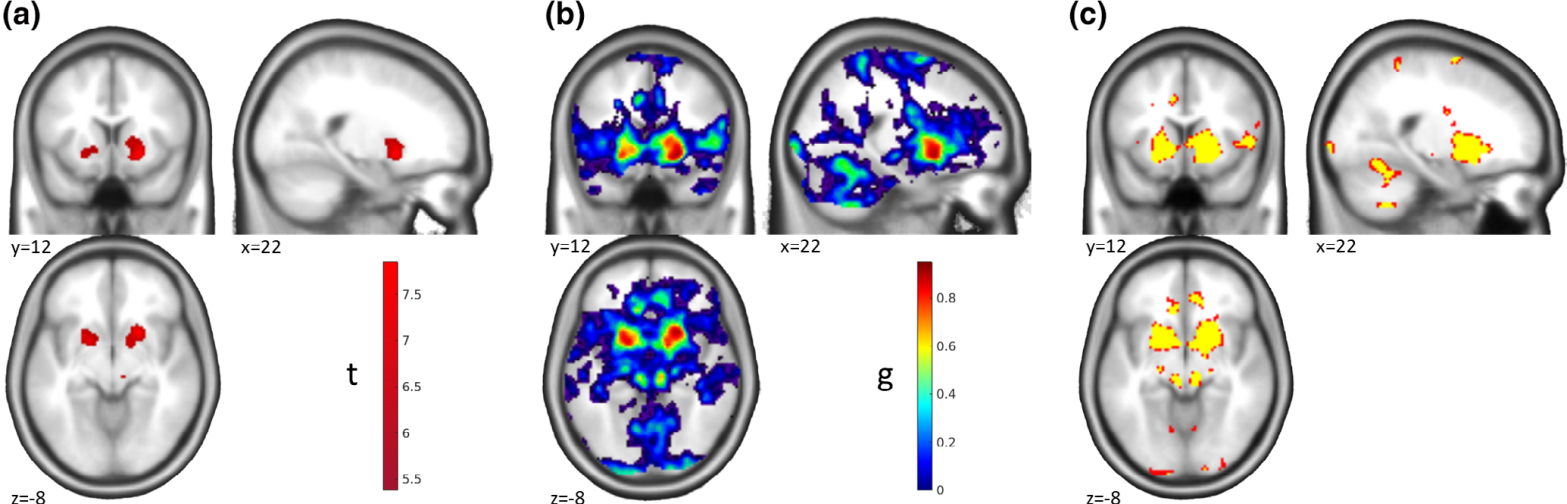
“Maps of Undecidability”—One‐Sample *t*‐test. Results for a monetary incentive delay task in a sample of *n* = 32 participants with Alcohol Use Disorder and *n* = 35 healthy controls. (a) Activation (*p* < .05 whole‐brain FWE corr.) for the main effect of the anticipation of monetary reward compared to the anticipation of verbal feedback in the whole sample showing a strong activation in bilateral striatum. (b) Map of ES *g* for the activations shown in (a). (c) Areas of undecidability in yellow are marking voxels for which ES 90% CI included the median effect size (*g* = 0.66) in the significant clusters. For comparison, uncorrected activation (*p* < .001 unc.) is shown in red. In this specific example the areas of undecidability are largely overlapping with the uncorrected activation and are just slightly more spatially restricted. Please note that this correspondence depends on the exact chosen reference value and the properties of the specific data set for a given analysis. Reanalyzed data from Becker et al. (2017)

**FIGURE 3:**
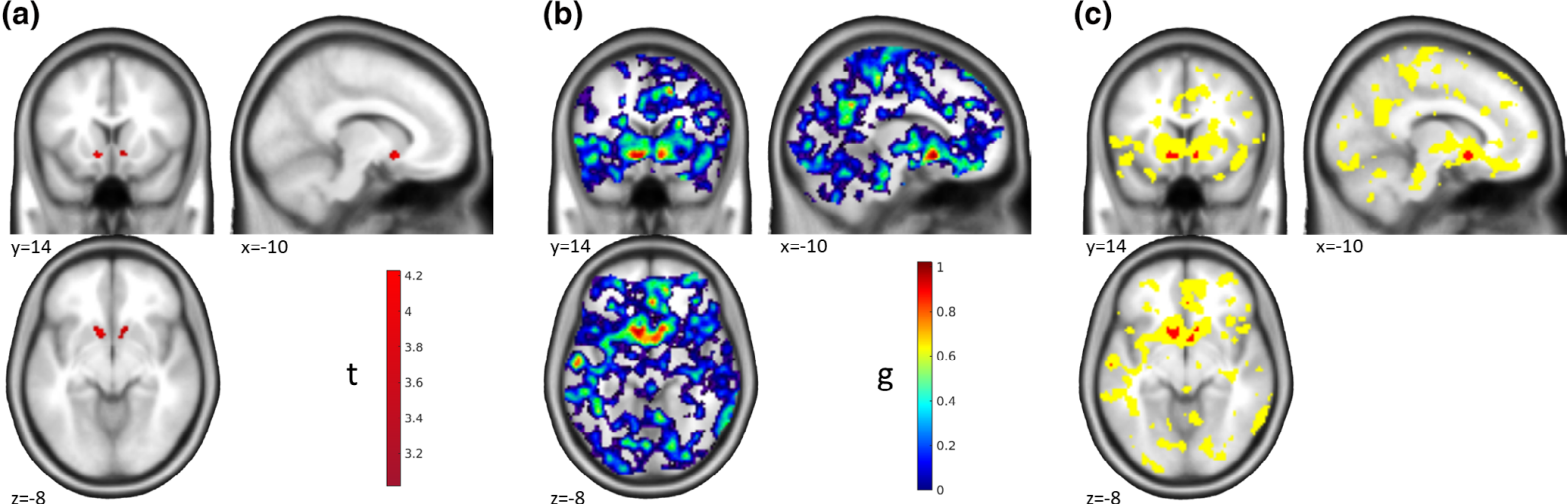
“Maps of Undecidability”—Two‐Sample *t*‐test. Results of the group comparison for the monetary incentive delay task comparing participants with Alcohol Use Disorder and healthy controls. (a) Activation for the group comparison (AUD > HC) based on ROI analyses in the left and right nucleus accumbens (*p* < .025 FWE ROI analyses in each of the two ROIs). Participants with Alcohol Use Disorder showed stronger reactions in the nucleus accumbens than healthy controls. See Becker et al. (2017) for further details and discussion. (b) Map of ES *g* for the activations shown in (a). (c) Areas of undecidability in yellow are marking voxels for which ES 90% CI included the median ES (*g* = 0.7289) in the significant clusters. For comparison, uncorrected activation (*p* < .001 unc.) is shown in red. In this example, the uncorrected activation is very restricted and the areas of undecidability are rather large and extend well beyond. Reanalyzed data from Becker et al. (2017)

We reanalyzed data from a monetary incentive delay task experiment performed by participants with Alcohol Use Disorder (AUD; *n*_AUD_ = 32) and healthy controls (HC; *n*_HC_ = 35) reported in Becker, Kirsch, Gerchen, Kiefer, and Kirsch (2017). We conducted analyses for the main effect (money > control) over both groups with a one‐sample *t*‐test (Figure 2) and for the group comparison (AUD > HC) with a two‐sample *t*‐test (Figure 3).

The one‐sample *t*‐test revealed a main effect of money > control in the bilateral ventral striatum at a threshold of *p* < .05 whole‐brain FWE corr. (Figure 2a); corresponding ES in Figure 2b). “Undecidable” regions were for example found in the anterior cingulate cortex (ACC), right insula, and cerebellum (yellow in Figure 2c). Interestingly, in this example and at the chosen reference ES the undecidable areas were largely consistent with, and only minimally smaller than, the effect at *p* < .001 unc. (red in Figure 2c).

In the group comparison, the picture was quite different. Here, we identified a more localized effect by ROI analyses in the left and right nucleus accumbens at a threshold of *p* < .025 FWE ROI analysis (*p* < .05 corrected for two hemispheres; Figure 3a; corresponding ES in Figure 3b). Here, this effect was largely consistent with the results at *p* < .001 unc. (red in Figure 3c), and the areas of undecidability extended well beyond to, for example, striatum, ACC, posterior cingulate cortex, and insula (yellow in Figure 3c), reflecting a high uncertainty in the analysis about the uniqueness of the apparently very local effect. This result makes sense given the lower power of between‐subjects comparisons to detect significant effects and corresponding larger confidence intervals of the effect sizes.

The comparison with *p* < .001 unc. in the two examples shows that there is no conceptual similarity of our approach with just using a more liberal statistical threshold, although the results might coincide in some cases.

### 3.2 Replication

Another directly apparent application of ES and their CIs in fMRI is testing for the replicability of detected effects. A voxel‐wise strategy can be applied either with a general reference ES or with a reference map. For demonstration, we reanalyzed a data set from an episodic memory task (Gerchen & Kirsch, 2017; see Supplement for further information) with two subsamples (*N* = 136; *n*_1_ = 54, *n*_2_ = 82) scanned with the same protocol at different sites. Reflecting the situation that might occur in a replication study we use the smaller sample as the reference data set and the larger sample as the replication set. Both samples were originally analyzed together with the same analysis pipeline, which enables us to conduct voxel‐wise comparisons. As replication criterion we tested whether the ES obtained with the reference sample fall into the voxel's ES 90% CI in the replication sample. Following the usual approach in fMRI, we focused on effects in one contrast direction (encoding > control), and restricted our analyses to voxels that had an ES *g* > 0 in the reference data set. Similar tests could be added for the opposite contrast direction.

**FIGURE 4:**
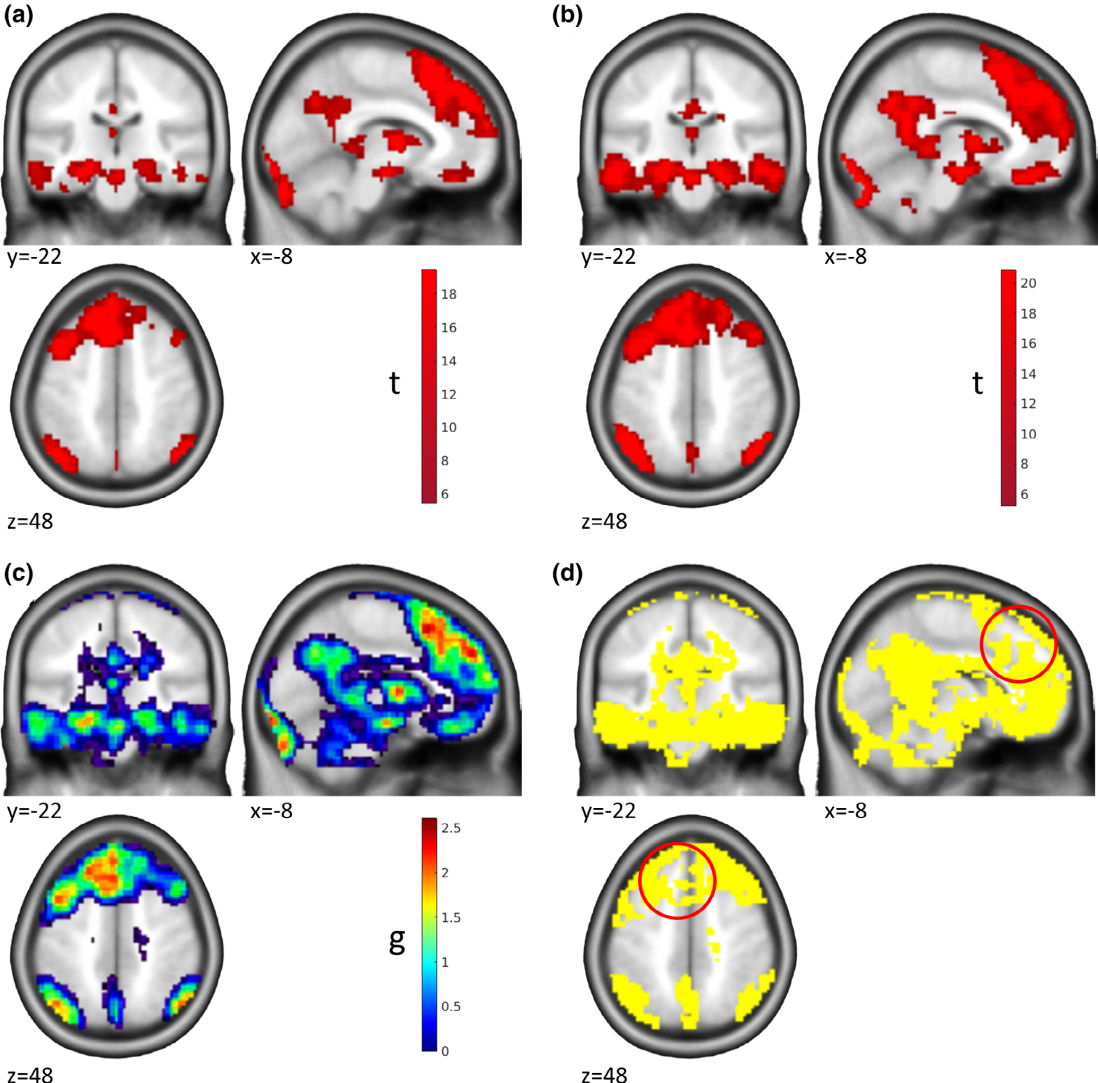
Replication of Effects. Results from the encoding phase of an episodic memory task are shown. (a) Activation (*p* < .05 whole‐brain FWE corr.) for the contrast encoding > control in the reference sample of *n*1 = 54 participants. (b) Activation (*p* < .05 whole‐brain FWE corr.) for the contrast encoding > control in the replication sample of *n*2 = 82 participants. Both samples were acquired in the same project with the same protocol but at different sites. (c) Map of ES *g* for the activations shown in (a). (d) Yellow marks voxels where the ES 90% CI in the replication sample includes the ES of the voxel in the reference sample, which we define as a replication of the original effect size. Red circles: Area where the effect was significant in both samples but the reference ES did not replicate. Please note that only voxels are shown where the reference effect size was *g* > 0. Data from Gerchen and Kirsch (2017)

*T* maps thresholded at *p* < .05 whole‐brain FWE corrected for the two samples are shown in Figure 4a,b, the ES map for the reference sample is shown in Figure 4c. The task leads to broadly distributed activations which are largely overlapping between the two samples. Interestingly, the voxel‐wise test reveals further details beyond the overlap of significant effects (Figure 4d). First, small ES were replicated in large areas where no significant effect was detected. More importantly, very large ES in the original sample failed to replicate (red circles in Figure 4d), although the voxels were detected as significant in both samples, suggesting that the initial ES estimates in these areas were biased in the positive direction and should thus not be taken as representative of the underlying effect. It is important to note that this information could not have been detected by NHST and concerns ES beyond plain statistical significance. It is a well‐described phenomenon that ES are declining over replications (see for example, Ioannidis, 2005a, 2005b; Open Science Collaboration, 2015), and this approach allows for formal testing of such phenomena in fMRI data. Importantly, the question whether or not a finding has replicated is far from trivial as is demonstrated by the five different definitions of replication success used by the Open Science Collaboration (2015): (1) statistical significance of the replication, (2) whether the 95% CI included the point estimate of the original study, (3) comparison of the original and the replication effect sizes, (4) meta‐analytical comparison of the effect sizes, and (5) subjective assessment by the researchers. Our approach allows researchers to assess replication by asking whether the 95% CI included the point estimate of the original study, but this must be weighed against other indicators of replication success.

### 3.3 Lateralization

An important question that arises in numerous contexts in functional neuroimaging is whether a detected effect is lateralized, that is, more pronounced in one of the two hemispheres. Often a lateralization index is calculated (see e.g., Bradshaw, Bishop, & Woodhead, 2017; Bradshaw, Thompson, Wilson, Bishop, & Woodhead, 2017), but formal testing is difficult. Inferiority tests against a reference ES representative for a detected cluster offer a straightforward approach to address this question.

**FIGURE 5:**
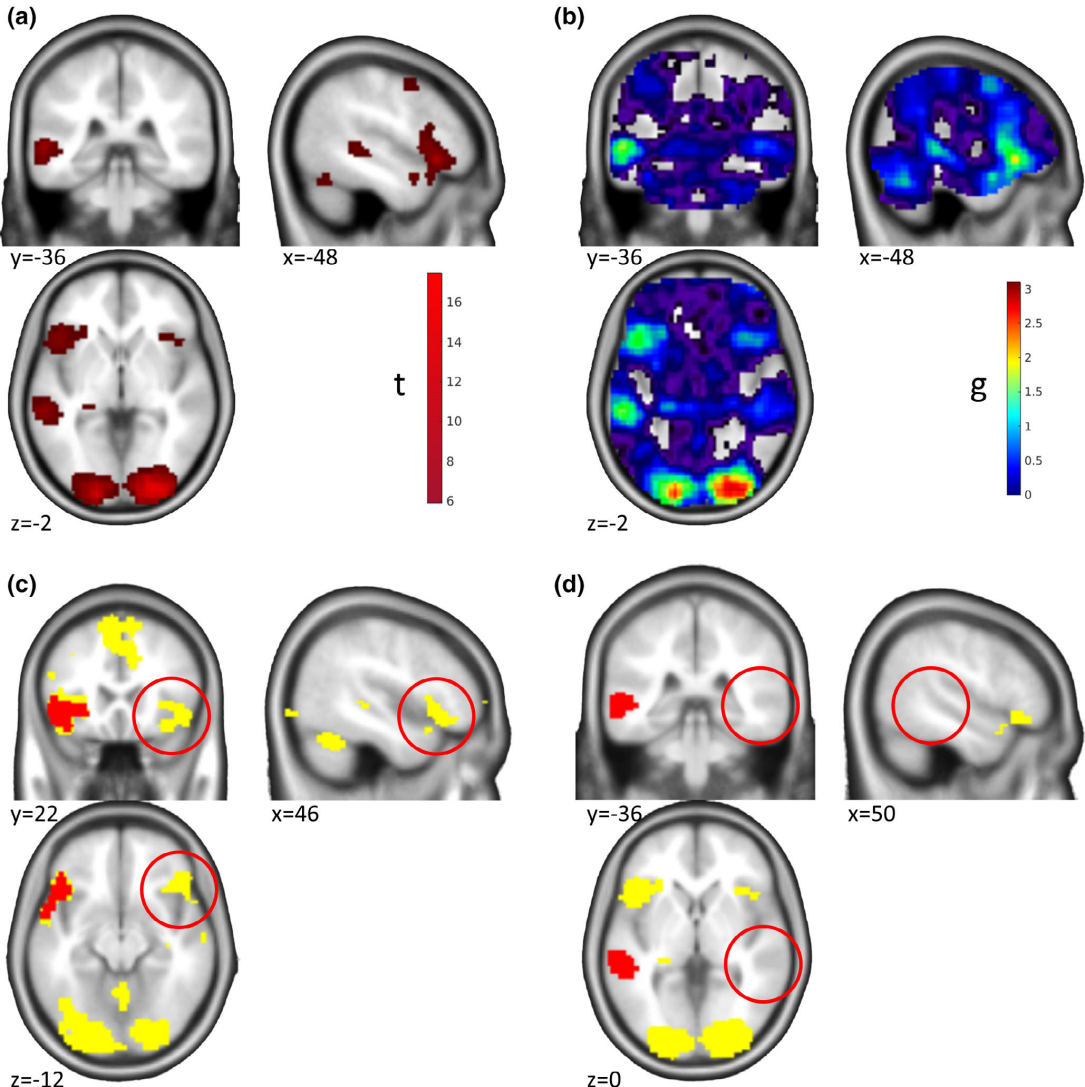
Lateralization of Effects. Results for a written statement presentation task are shown. (a) Activation (*p* < .05 whole‐brain FWE corr.) for the main effect of written statement presentation in *N* = 30 healthy participants. (b) Map of ES *g* for the activations shown in (a). (c) Regions (yellow) that cannot be assumed to be smaller than the 75th percentile ES in the reference cluster (red) including Broca's area. Red circles: Area in the right inferior frontal gyrus, suggesting contralateral effects in our data that cannot be shown to have a smaller effect than the reference cluster. (d) Regions (yellow) that cannot be assumed to be smaller than the 75th percentile ES in the reference cluster (red) in the left ventral occipito‐temporal cortex. Red circles: Inferior effects in the corresponding right left ventral occipito‐temporal cortex, suggesting lateralization of effects in our data. Unpublished data by M.F. Gerchen

As an example, we use unpublished data from a statement judgment task where short written statements were presented to healthy right‐handed participants (*N* = 30) and rated as true or false (See Supplement for further information). Here, we did not focus on any specific experimental effect but analyzed the main effect of sentence presentation, which, beside others, showed strong activation in the left ventral occipito‐temporal cortex and Broca's area (Figure 5a,b) associated with language processing (e.g., Bradshaw, Thompson, et al., 2017). Language processing has traditionally been described as lateralized to the left hemisphere in right‐handed individuals (e.g., Bradshaw, Thompson, et al., 2017). Thus, we tested for these two clusters whether comparable effects are present in contralateral areas. For this we selected a reference ES reflecting a rather strong activation in the detected cluster and take the voxel with the 75th percentile ES in the reference cluster as the criterion. In other words, we test whether voxels whose ES 90% CI upper limit exceeds or includes the 75th percentile ES in the reference cluster are present in the respective contralateral region.

For the FWE corrected significant reference clusters, the 75th percentile ES was *g* = 1.3242 for left Broca's area, and *g* = 1.454 in the left superior temporal cluster. Voxels with 90% ES CIs including the respective reference ES could be identified in the right inferior frontal cortex (Figure 5c), but not in the left ventral occipito‐temporal cortex (Figure 5d). These results demonstrate how ES and their CIs can be used to provide evidence for, as well as against, lateralization in fMRI studies.

## 4 DISCUSSION

In this paper, we described the construction of the standardized ES Hedges' *g* and its CI for *t*‐tests in statistical parametric mapping and demonstrated in selected examples how these can be used to identify “regions of undecidability”, to conduct voxel‐wise replication tests, and for formal testing of lateralization of effects. Overall, our examples further demonstrate that NHST alone does not provide a conclusive picture about the effects contained in fMRI data, especially about the equivalence or inferiority of effects, and that complementary analyses as described allow important further insight into the results. Importantly, by sharing only the group level statistics (i.e., the t map and design matrix) for a specific contrast researchers can enable other researchers to apply these analyses without having to grant access to the raw data.

Obviously, a central decision for the described procedures with strong influence on the results and conclusions is the selection of the reference ES. Therefore, it is of uttermost importance that this selection is made a priori based on justifiable reasons related to the goal of the analysis and preregistered before the analysis is conducted. Within a data set, a number of possible criteria are for example the minimum, maximum, median, or quantile effect sizes in a reference cluster. As the ES are estimated in a voxel‐wise manner, it might not be advisable to choose the mean or other summarizing values here.

It should be noted that very large sample sizes would lead to high precision and small CIs, and thus would for example reduce the number of replicated effects in the “replication” example. This might, however, be regarded as a somewhat ideal situation because the discussion could then focus on the meaning of the detectable effect size differences and the real underlying effect size, overcoming several of the well‐known shortcomings of NHST.

It is further important to note that the smaller the reference ES (and the larger the CI) gets, the larger are the areas with overlapping CIs. It depends on the goal of the intended analysis what determines a liberal or conservative procedure.

In our examples, we did not use a correction for multiple comparisons because such a correction has more complex consequences than in NHST and a more conservative thresholding can work in favor or against the interpretation a researcher might prefer. In general, what a multiple comparison correction algorithm would do is to widen the confidence interval (corresponding to a smaller statistical threshold) to ensure that the overall level of confidence is controlled. In our “maps of undecidability” analyses, a widening of the confidence interval would lead to more voxels becoming non‐separable from the detected significant results, and thus would enlarge the undecidable areas. The reported uncorrected results are therefore representing the lower bound of the size of the undecidable regions. In the “replication” analysis, a widened CI would lead to less voxels being identified as “not replicated”. Stricter control of false positives would thus have the somewhat contradictory effect to increase the replicability of effects. In the “lateralization” sample, a widened CI would make it more likely to identify contralateral effects where the reference effects falls into the CI, while it would be more difficult to demonstrate lateralized effects. Because we are demonstrating both directions in our example, we also report uncorrected results here.

Our paper is closely related to the work of Bowring et al. (2021) who developed an approach to identify confidence sets based on Cohen's *d* to identify brain regions with effects above and below a specified effect size. While we derive Hedges'g and its CI in a generalized way from the SPM GLM and thus cover in principle all cases for which SPM provided t values, Bowring et al. (2021) emphasize on implementing multiple comparison correction for one‐sample *t*‐tests by a Wild t‐Bootstrap procedure which controls the overall level of false positives in their confidence sets. A combination of the approaches might thus provide a way to implement equivalence and inferiority tests in fMRI with multiple comparison correction.

In the “maps of undecidability” approach, we are selecting a reference value within a data set. This is however no circular analysis because this procedure is not biasing the analyses in the remaining part of the brain where the relevant comparisons of interest are conducted. However, when using such a data driven approach to choose the reference value, we advocate strongly for precisely documenting the selection procedure via preregistration to prevent biasing the results.

If a data set should be used for replication of an external effect from another study, our procedures would for example allow to implement a voxel‐wise small telescope approach in which one could tests for the existence of effects that an original study could have meaningfully examined (Simonsohn, 2015). Another interesting application for our method is using a smallest effect size of interest (SESOI) as the threshold (Lakens et al., 2018). In practice, it may be hard to determine the SESOI, but in large samples, it would allow excluding those voxels that have a significant activation that is too small to matter conceptually.

While our treatment covers only Hedge's *g* as a specific ES for *t*‐tests in SPM, these tests nonetheless cover a substantial part of analyses in the fMRI field. If other ES are needed, the MES toolbox (Hentschke & Stüttgen, 2011) provides a comprehensive library of ES and their CIs, which could be adapted for fMRI data in a similar way as we demonstrated here. Overall, we strongly believe the field of neuroimaging will benefit from providing evidence for absence of effects as much as for their presence and we hereby provide a method using a NHST‐approach that can complement other approaches such as Bayesian statistics. Since this method is applied at the group level, data to perform these analyses can be shared without invoking data protection issues. Sharing t maps and design matrices of every effect that is reported in a paper would thus enable other researchers to use this method, for example, to scrutinize the robustness of the reported focal effect.

## CONFLICT OF INTEREST

The authors declare that they have no known competing financial interests or personal relationships that could have appeared to influence the work reported in this paper.

## ETHICS STATEMENT

All procedures conformed to the Declaration of Helsinki and were approved by the local ethics committees of the Medical Faculties of Heidelberg University.

## Supporting information


**APPENDIX S1**: Supporting Information

## Data Availability

The fMRI raw data for the examples cannot be made publicly available due to protection of sensitive personal information. The Matlab script to estimate effect sizes and their CI from SPM t maps, the script for simulations in Figure 1 as well as the second level t maps, design matrices, and contrast vectors for the reported example data are available on Github at https://github.com/Fungisai/g_ci_spm.
