# Supplementary material for: Brain‐wide inferiority and equivalence tests in fMRI group analyses: Selected applications
Source: Hum Brain Mapp. 2021 Sep 16;42(18):5803–13. doi: 10.1002/hbm.25664 (PMC8596945; doi:10.1002/hbm.25664)
Supplement: Supplementary file 1 — APPENDIX S1: Supporting Information [file HBM-42-5803-s001.docx]

**Brain-Wide Inferiority and Equivalence Tests in fMRI Group Analyses:**

**Selected Applications**

Martin Fungisai Gerchen^1,2,3^, Peter Kirsch^1,2,3^, & Gordon Benedikt Feld^1,3,4,5^

^1^Department of Clinical Psychology, Central Institute of Mental Health, Medical Faculty Mannheim, Heidelberg University, Mannheim, Germany

^2^Bernstein Center for Computational Neuroscience Heidelberg/Mannheim, Mannheim, Germany

^3^Department of Psychology, Heidelberg University, Heidelberg, Germany

^4^Department of Addiction Behavior and Addiction Medicine, Central Institute of Mental Health, Medical Faculty Mannheim, Heidelberg University, Mannheim, Germany

^5^Department of Psychiatry and Psychotherapy, Central Institute of Mental Health, Medical Faculty Mannheim, Heidelberg University, Mannheim, Germany

**Supporting Information**

**Data simulation**

We simulated one slice of fMRI data for a control group and an experimental group by setting up two 2d matrices with 0 for all voxels except for the three points indicated (I, II, III). For these points we chose the effect sizes of 0.45 (I), 0.8 (II) and 0.8 (III) for the experimental group’s matrix only. Next, we applied a spatial Gaussian filter, so that the effects at the three points were spread out spatially, which reduced the maxima to 0.28 (I), 0.50 (II) and 0.50 (III), i.e., the true underlying effects at those points. We then pulled random numbers from a normal distribution for each simulated participant (N = 500 per group) using the numbers from the 2d matrices as mean per voxel and a standard deviation of 1 (therefore the simulated effects correspond to Cohen’s d). We did not constrain our simulation anatomically, so no structural alignment should be expected. For Figure 1 we either analyzed the complete simulated data (b) or only a sub sample (c).

**Supplemental Materials & Methods**

*“Maps of Undecidability” Analysis*

We reanalyzed data from Becker et al. (2017). The sample included n_AUD_= 32 participants with a diagnosis of Alcohol Use Disorder (Age 45.4±9 years; 77.4% male) recruited from the Central Institute of Mental Health inpatient addiction clinic and n_HC_=35 healthy control participants (Age 47.7±8.9 years; 65.7% male) matched for age, sex, and education recruited from the local population. Patients were abstinent for 11±5.6 days at the time of the experiment. All participants provided fully informed written consent and all procedures conformed to the Declaration of Helsinki and were approved by the local ethics committee of the Medical Faculty Mannheim of Heidelberg University.

In the experiment participants performed a monetary incentive delay reaction-time task (Kirsch et al. 2003). In this task participants have to react by pressing a button as fast as possible when a flash occurs. In each trial they are first informed by a cue about the consequences of a fast response (monetary reward: gain 2€; punishment avoidance: avoidance of a loss of 2€; verbal feedback: feedback on the performance; passive control condition: no response is required) which is shown until the flash occurs after 6s. After the response the participants see the respective feedback. The reaction time criterion is flexibly adapted to the reaction time of the participants. After the experiment participants received the money they had won in cash.

Magnetic resonance imaging was conducted with a 3T Siemens Trio scanner (Siemens Healthineers, Erlangen, Germany) with a 12-channel head coil. T1-weighted anatomical images (MPRAGE) were collected with TR=2.3s, TE=3.03ms, flip angle 9° in 192 sagittal slices with slice thickness 1.0mm, in-plane resolution of 1mmx1mm and field of view FoV=256×256mm. 267 functional images were acquired with an echo-planar imaging (EPI) sequence with TR=2s, TE=30 ms, flip angle 80° in 28 slices with slice thickness 4.0 mm with 1mm gap and matrix size = 64×64.

Analyses were conducted with SPM8 (v4515) in MATLAB R2011b. The functional images were slice-time corrected, realigned, co-registered to the anatomical image, normalized into MNI space and smoothed with a 6mm full-width at half-maximum Gaussian kernel. The ART toolbox (<http://www.nitrc.org/projects/artifact_detect>) was used to identify motion-affected volumes with volume-to-volume movements >1mm and global intensity changes z>7.

First-level analyses were conducted with general linear models including the event-related condition regressors convolved with the canonical HRF, 6 conventional motion parameters, white matter (WM) and cerebrospinal fluid (CSF) signals and the ART dummy regressors as covariates. A high-pass filter with a cut-off period of 128s was applied. Only the contrast monetary reward > verbal feedback was used and the contrast maps of the participants were used for the second-level analysis. A one sample t-test was conducted for the main effect of monetary reward > verbal feedback at a significance threshold of p<0.05 whole-brain FWE corr. ROI analyses in the left and right nucleus accumbens at a threshold of p<0.025 FWE ROI corr. (p<0.05 corrected for two hemispheres) was conducted with a two sample t-test to test for group differences in the direction AUD>HC. In contrast to the analyses reported in Becker et al. (2017) we used here a slightly more robust procedure to conduct the ROI analyses but come to the same results.

*Replication Analysis*

We reanalyzed data from Gerchen & Kirsch (2017). The data set comprised two samples with n_1_=54 and n_2_=82 healthy right-handed participants. The average age in the whole sample was 31.99±9.72 years and 47.8% of the participants were female.

In the experiment participants performed an episodic memory task (Erk et al., 2010) in which they learned associations between faces and professions. Here, we analyzed only data from the encoding phase. In this phase participants saw 16 face-profession pairs (each twice) in an ABAB block design with 4 experimental and 6 control blocks and were instructed to remember the associations. During face presentation participants had to indicate by a left or right button press whether faces and professions were matching well in their own opinion. During the control blocks schematic heads were shown and the participants indicated which of the two ears was larger.

All participants provided fully informed written consent and all procedures conformed to the Declaration of Helsinki and were approved by the local ethics committees of the Medical Faculties Mannheim and Heidelberg of Heidelberg University.

Magnetic resonance imaging was conducted with two similar 3T Siemens Trio scanners (Siemens Healthineers, Erlangen, Germany) at the Central Institute of Mental Health Mannheim and at the University of Heidelberg, Germany. 244 functional images were acquired with echo-planar imaging (EPI) with TR=1.8s, TE=30ms, flip angle 73°, in 33 slices with slice thickness 3.0 mm with 1mm gap, a field of view of FoV=192 mm, and GRAPPA with iPAT=2.

Analyses were conducted with SPM8 (v4667) in MATLAB R2011b. The functional images were slice-time corrected, realigned, normalized into MNI space, rescaled to 3x3x3mm, and smoothed with a 6mm full-width at half-maximum Gaussian kernel. The ART toolbox was used to identify motion-affected volumes with volume-to-volume movements >0.5mm and global intensity changes z>6.

First-level analyses were conducted with general linear models including the block regressors convolved with the canonical hemodynamic response function (HRF), 6 conventional motion parameters, the CSF signal and the ART dummy regressors as covariates. A high-pass filter with a cut-off period of 128s was applied. Only the contrast encoding > recall was used and the contrast maps of the participants were used for the second-level analysis which were conducted with one sample t-tests within each subsample with age and sex as covariates. A significance threshold of p<0.05 whole-brain FWE corr. was applied.

*Lateralization Analysis*

For the lateralization analysis we used unpublished data from a written sentence presentation and judgement task by M.F. Gerchen. The sample consisted of N=30 healthy right-handed participants (age 23.37±1.43 years; 73.3% female) who were mainly university students. In the task short written statements with a maximum of 10 words from the categories religion, facts, conspiracy theories, superstition, and politics were presented for a duration of 6.56-8.2s in 100 trials. After each statement participants answered the forced-choice question whether they think the statement is true or not with a button press and rated their certainty on a visual analog scale from 0-100%. All participants provided fully informed written consent and all procedures conformed to the Declaration of Helsinki and were approved by the local ethics committee of the Medical Faculty Mannheim of Heidelberg University (2019-724N).

Magnetic resonance imaging was conducted with a 3T Siemens Trio scanner (Siemens Healthineers, Erlangen, Germany). T1-weighted anatomical images (MPRAGE) were collected with TR=2.3s, TE=3.03ms, flip angle 9° in 192 sagittal slices with slice thickness 1.0mm, in-plane resolution of 1mmx1mm and field of view FoV=256×256mm. Functional images were acquired with an echo-planar imaging (EPI) sequence with TR=1.64s, TE=30ms, flip angle 80° in 30 slices with slice thickness 3.0 mm with 1mm gap with 3x3mm in-plane resolution, and GRAPPA with iPAT=2. The duration of the experiment depended on the response speed and the average scan duration was 866.1±65.82 volumes. During scanning pulse and respiration were monitored and saved.

Analyses were conducted with SPM12 (v7219) in MATLAB R2017a. The anatomical image was segmented and normalized to MNI space. The functional images were slice-time corrected, realigned, co-registered to the anatomical image, normalized into MNI space, rescaled to 3x3x3mm resolution and smoothed with an 8mm full-width at half-maximum Gaussian kernel. The ART toolbox was used to identify motion-affected volumes with volume-to-volume movements >0.5mm and global intensity changes z>4. The TAPA PhysIO toolbox (Kasper et al., 2017) was used to estimate physiological nuisance regressors.

First-level analyses were conducted with general linear models including the event-related condition regressors convolved with the canonical HRF, 6 conventional motion parameters, WM, CSF, and whole-brain signals were used as covariates along with the ART dummy regressors and the PhysIO physiological nuisance regressors. A high-pass filter with a cut-off period of 128s was applied. Here we only analyzed the main effect of written sentence presentation. The reported analyses were not intended when the study was planned and are not part of the main results of the study. The contrast maps of the participants were used for second-level analysis with a one sample t-test in which age and sex were included as covariates. A significance threshold of p<0.05 whole-brain FWE corr. was applied.

**References**

Becker, A., Kirsch, M., Gerchen, M. F., Kiefer, F., & Kirsch, P. (2017, May). Striatal activation and frontostriatal connectivity during non-drug reward anticipation in alcohol dependence. *Addict Biol, 22*(3), 833-843. https://doi.org/10.1111/adb.12352

Erk, S., Meyer-Lindenberg, A., Schnell, K., Opitz von Boberfeld, C., Esslinger, C., Kirsch, P., Grimm, O., Arnold, C., Haddad, L., Witt, S. H., Cichon, S., Nothen, M. M., Rietschel, M., & Walter, H. (2010, Aug). Brain function in carriers of a genome-wide supported bipolar disorder variant. Arch Gen Psychiatry, 67(8), 803-811. https://doi.org/10.1001/archgenpsychiatry.2010.94

Gerchen, M. F., & Kirsch, P. (2017, Nov). Combining task-related activation and connectivity analysis of fMRI data reveals complex modulation of brain networks. *Hum Brain Mapp, 38*(11), 5726-5739. https://doi.org/10.1002/hbm.23762

Kasper, L., Bollmann, S., Diaconescu, A. O., Hutton, C., Heinzle, J., Iglesias, S., Hauser, T. U., Sebold, M., Manjaly, Z. M., Pruessmann, K. P., & Stephan, K. E. (2017, Jan 30). The PhysIO Toolbox for Modeling Physiological Noise in fMRI Data. *J Neurosci Methods, 276*, 56-72. https://doi.org/10.1016/j.jneumeth.2016.10.019
